# Immune checkpoint inhibitor-related thyroid dysfunction during treatment of lung cancer: a disproportionality analysis based on the FDA adverse event reporting system

**DOI:** 10.3389/fimmu.2026.1902939

**Published:** 2026-09-09

**Authors:** Yaqian Tan, Qi Song

**Affiliations:** 1 Department of Pharmacy, The Affiliated Brain Hospital, Guangzhou Medical University, Guangzhou, China; 2 Key Laboratory of Neurogenetics and Channelopathies of Guangdong Province and The Ministry of Education of China, Guangzhou Medical University, Guangzhou, China; 3 Department of Pharmacy, Guangzhou Institute of Cancer Research, the Affiliated Cancer Hospital, Guangzhou Medical University, Guangzhou, China

**Keywords:** adverse event, FAERS, immune checkpoint inhibitors, pharmacovigilance, thyroid dysfunction

## Abstract

**Background:**

Immune checkpoint inhibitors (ICIs) have exhibited remarkable clinical benefits in the treatment of lung cancer, yet a large-scale pharmacovigilance investigation on ICI-related thyroid dysfunction (ICI-TD) remained limited. This study aimed to comprehensively analyze the demographic characteristics of ICI-TD in lung cancer patients and the pharmacovigilance signal patterns of the adverse events (AEs) using data from the Food and Drug Administration Adverse Event Reporting System (FAERS) database.

**Methods:**

All existing data ranging from January 1^st^ 2014 to March 31^st^ 2026 were retrieved from the FAERS database. In descriptive analysis, the demographic characteristics of ICI-TD in lung cancer patients were collected and categorized. Disproportionality analysis was conducted to evaluate the signal characteristics of ICI-TD by employing three disproportional algorithms, namely reporting odds ratio (ROR), proportional reporting ratio (PRR), and information component (IC).

**Results:**

A total of 2,001 reports of ICI-TD during treatment of lung cancer were identified after data processing, involving 1,758 patients and 10 ICIs. In general, ICI-TD were mainly reported in male patients (n = 959, 54.55%) and patients aged ≥ 65 years (n = 856, 48.69%; median [lower quartile (Q1), upper quartile (Q3)] = 67 [60, 73]). After data cleaning, we found that reports from 752 patients had valid time-to-onset (TTO) values. The median TTO of ICI-TD was 42 days [Q1, Q3] = [14, 98], and ICI-TD was predominantly reported within 30 days after ICI administration (n = 323, 42.95% of all 752 patients). The vast majority of patients were reported as having serious reaction outcome (n = 1,690, 96.13%). Among all ICIs, nivolumab had the greatest number of ICI-TD reports (a = 655, 32.73% of all 2,001 reports, ROR_025_ = 2.97, IC_025_ = 1.32, PRR = 3.21, χ^2^ = 782.34). The most frequently reported ICI-TD was “hypothyroidism” (a = 901, 45.03% of all 2,001 reports, ROR_025_ = 4.83, IC_025_ = 1.22, PRR = 5.36, χ^2^ = 1,138.77).

**Conclusions:**

The present study provided a comprehensive overview of the demographic features of patients and pharmacovigilance signal patterns of ICI-TD during the treatment of lung cancer. Our findings would offer a referential perspective for the clinical understanding of the pharmacovigilance signals of ICI-TD.

## 1 Introduction

Lung cancer is a highly malignant tumor that ranks first with a percentage of 18% among all cancer-related deaths, posing substantial challenges to the public health systems worldwide (1, 2). Various treatment strategies for lung cancer have been applied in clinic, including surgery, chemotherapy, radiotherapy, and targeted therapy (3). Over the last decade, the discovery of immunotherapy has brought significant breakthrough in the therapies of various tumor types, such as lung cancer, melanoma, head and neck squamous cell cancer, urothelial cancer, and hepatocellular carcinoma (4). In immunotherapy, the application of immune checkpoint inhibitors (ICIs) strengthen the immune responses against tumor cells at different stages by targeted suppression of immune checkpoint molecules, which significantly improve the therapeutic effect of cancer (5, 6). According to the different targets of action, ICIs are mainly divided into three categories, including anti-programmed cell death receptor-1 (PD-1), anti-programmed death ligand-1 (PD-L1), and anti-cytotoxic T-lymphocyte-associated antigen 4 (CTLA-4) (7).

Although ICIs have brought significant clinical efficacy, their intrinsic mechanism on the modification of immune tolerance unavoidably trigger ICI-related adverse events (AE), predominantly impacting the digestive system, respiratory system, skin, and endocrine system (8). Notably, ICI-related endocrine AEs are characterized by their irreversible nature and the need for lifelong hormone replacement therapy, thereby posing clinical challenges (9). The commonly observed ICI-related endocrine toxicities include thyroid dysfunction, adrenal insufficiency, and hypophysitis (10). Among them, ICI-related thyroid dysfunction (ICI-TD) is the most common AE, mainly presenting as hypothyroidism, hyperthyroidism, and thyroiditis (11). Due to the expanding application of ICIs in the treatment of lung cancer, it is therefore essential to systematically analyze a large-scale post-marketing dataset to investigate the clinical characteristics of ICI-TD.

So far, there have been several studies that mined the safety profiles of ICI-TD from various spontaneous AE reporting databases (10, 12, 13). However, differences in study scopes (e.g., drug inclusion, AE inclusion, time window, and indication) mean that the evidence still needs to be further enriched. Other studies focusing on ICI-TD were mostly originated from clinical trials and meta-analysis (14–17), and lacked a perspective from post-marketing surveillance data. The United States FDA Adverse Event Reporting System (FAERS) database is the largest voluntary AE reporting system worldwide that has been broadly recognized as a drug surveillance tool (18, 19). Therefore, the purpose of this research was to carry on a disproportionality analysis to identify the demographic characteristics and pharmacovigilance signal patterns of ICI-TD in patients with lung cancer using data from the FAERS database. Our findings would offer a referential perspective for the clinical understanding of the pharmacovigilance signals of ICI-TD.

## 2 Materials and methods

### 2.1 Data source and study design

We conducted a pharmacovigilance study of ICI-TD in patients with lung cancer using all existed individual case safety reports in the FAERS database (https://www.fda.gov/drugs/drug-approvals-and-databases/fda-adverse-event-reporting-system-faers-database), covering the timeframe from January 1^st^ 2014 to March 31^st^ 2026. All data extraction was performed on a single day of August 4^th^ 2026. In the formal analysis, a total of seven categories of retrieved files were used, including demographic and administrative data (DEMO), drug information (DRUG), adverse drug reactions (REAC), patient outcomes (OUTC), therapy start and end dates (THER), report sources (RPSR), and indications (INDI).

The drug names of the target ICIs were standardized into generic names, and the detailed list was as follows: PD-1 (cemiplimab, nivolumab, pembrolizumab, tislelizumab, and toripalimab), PD-L1 (atezolizumab, avelumab, and durvalumab), and CTLA-4 (ipilimumab and tremelimumab). Reports were included only when the ICIs were listed as “primary suspect” to ensure clear drug-AE association. Reports in which the ICIs were listed as “secondary suspect” or “concomitant” were excluded from the analysis to minimize confounding.

Since there is no specific standardized Medical Dictionary for Regulatory Activities (MedDRA) query for ICI-TD, we referred to the preferred terminology (PT) in the MedDRA (Version 29.0) and the validated PT list of ICI-TD in previous literature (10, 20, 21), and excluded the PTs that do not directly manifest as ICI-TD. The final 16 PTs of ICI-TD included “atrophic thyroiditis”, “autoimmune hypothyroidism”, “autoimmune thyroid disorder”, “autoimmune thyroiditis”, “hyperthyroidism”, “hypothyroid myopathy”, “hypothyroidism”, “immune-mediated hyperthyroidism”, “immune-mediated hypothyroidism”, “immune-mediated thyroiditis”, “thyroid atrophy”, “thyroid dermatopathy”, “thyroid disorder”, “thyroid hormones decreased”, “thyroiditis”, and “thyrotoxic crisis”.

This study aimed to identify the pharmacovigilance metrics of ICI-TD in patients with lung cancer, and the items related to lung cancer from the INDI file were selected, including “adenosquamous cell lung cancer”, “large cell lung cancer”, “lung adenocarcinoma”, “lung cancer”, “lung carcinoma cell type unspecified”, “lung neoplasm”, “lung neoplasm malignant”, “lung squamous cell carcinoma”, “neuroendocrine tumor of the lung”, “non-small cell lung cancer”, “non-small cell lung cancer”, “sarcomatoid carcinoma of the lung”, “small cell lung cancer”, and “squamous cell carcinoma of lung”.

This study strictly followed the FDA official guidance for data cleaning. Specifically, the “PRIMARYID”, “CASEID”, and “FDA_DT” fields from the DEMO file were selected, and sorted by the following order: “CASEID”, “FDA_DT”, and “PRIMARYID”. For reports with the same “CASEID”, the one with the largest “FDA_DT” value (i.e. the most recent report version) was maintained. If reports have the same “CASEID” and “FDA_DT”, the one with the largest “PRIMARYID” value was selected.

The time-to-onset (TTO) of AE was calculated as the duration between the date of AE and the start date of ICI treatment using the formula: TTO = Event time (“EVENT_DT” in DEMO file) - Start time (“START_DT” in THER file). If more than one TTO values were detected under a single CASEID, the one with the largest TTO value (i.e., the earliest onset of AE) was retained. During TTO data cleaning process, missing entries (e.g. “START_DT” or “EVENT_DT”) or input errors (e.g. negative TTO value calculated) were removed from further analysis. The age of patients and TTO values were assessed using median with interquartile range of lower quartile (Q1) and upper quartile (Q3).

### 2.2 Statistical analysis

The pharmacovigilance signals of ICI-TD were assessed using three disproportionality analysis methods, including reporting odds ratio (ROR), proportional reporting ratio (PRR), and Bayesian confidence propagation neural network (BCPNN). All algorithms were calculated based on a 2 × 2 contingency table (**Table 1**), with detailed mathematical formulae and significant criteria listed in **Table 2**. In the ROR method, a pharmacovigilance signal was deemed significant when the report number a ≥ 3 and the lower bound of the 95% confidence interval (CI) of ROR (ROR_025_) exceeded 1 (22). In the BCPNN approach, the information component (IC) was considered as a measure of this algorithm, and IC was determined significant when both the report number and lower bound of the 95% CI of IC (IC_025_) are greater than 0 (23). While in the PRR algorithm, a significant signal was considered if the report number a ≥ 3, PRR ≥ 2, and the value of chi-squared (χ^2^) ≥ 4 (24). To ensure the robustness of signal detection and minimize the bias caused by single algorithm, AEs were considered significant only when identified by all three algorithms. Data analysis was conducted with SAS software (version 9.4) and visualization was performed using GraphPad Prism (version 9.3.1). All methods and findings of this study were conducted based on the guideline of “REporting of A Disproportionality analysis for drUg Safety signal detection using individual case safety reports in PharmacoVigilance (READUS-PV)” (25, 26), and the READUS-PV checklist was provided in **Supplementary Table 1**.

**Table 1:** The 2 × 2 contingency table of disproportionality analysis.

Parameters	Target AEs	Other AEs	Total
Target drugs	a	b	a+b
Other drugs	c	d	c+d
Total	a+c	b+d	a+b+c+d

All data used in the contingency table were restricted to reports with lung cancer as the indication.

AEs, adverse events; a, number of reports containing both the target drug (ICIs) and target adverse drug reaction (ICI-TD); b, number of reports containing other adverse drug reaction of the target drug; c, number of reports containing the target adverse drug reaction of other drugs; d, number of reports containing other drugs and other adverse drug reactions.

**Table 2:** Algorithms, formulas, and positive signal criteria of disproportionality analysis.

Algorithms	Formulas	Criteria
ROR	*ROR =* $\frac{ad}{bc}$	ROR_025_ > 1, a ≥ 3
	*95%CI =* $e^{ln(ROR)\pm 1.96}\;\sqrt{\frac{1}{a}+\frac{1}{b}+\frac{1}{c}+\frac{1}{d}}$	
BCPNN	*IC =* $\log_{2}\frac{a(a+b+b+d)}{\left(a+b)(a+c\right)}$	IC_025_ > 0, a > 0
	*IC_025_ =* $IC-2\;\sqrt{V(IC)}$	
PRR	*PRR =* $\frac{a/(a+b)}{c/(c+d)}$	χ^2^ ≥ 4, PRR ≥ 2, a ≥ 3
	χ^2^ *=* $\Sigma [{\left(O-E\right)}^{2}/E]$ *O =* a, *E =* $\frac{\left(a+b)(a+c\right)}{a+b+c+d}$	

ROR, reporting odds ratio; ROR _025_, the lower limit of the 95% CI of the ROR; 95% CI, 95% confidence interval; BCPNN, Bayesian confidence propagation neural network; IC, information component; IC_025_, the lower limit of the 95% CI of the IC; PRR, proportional reporting ratio; χ^2^, chi-squared.

## 3 Results

### 3.1 Descriptive analysis

**Figure 1:**
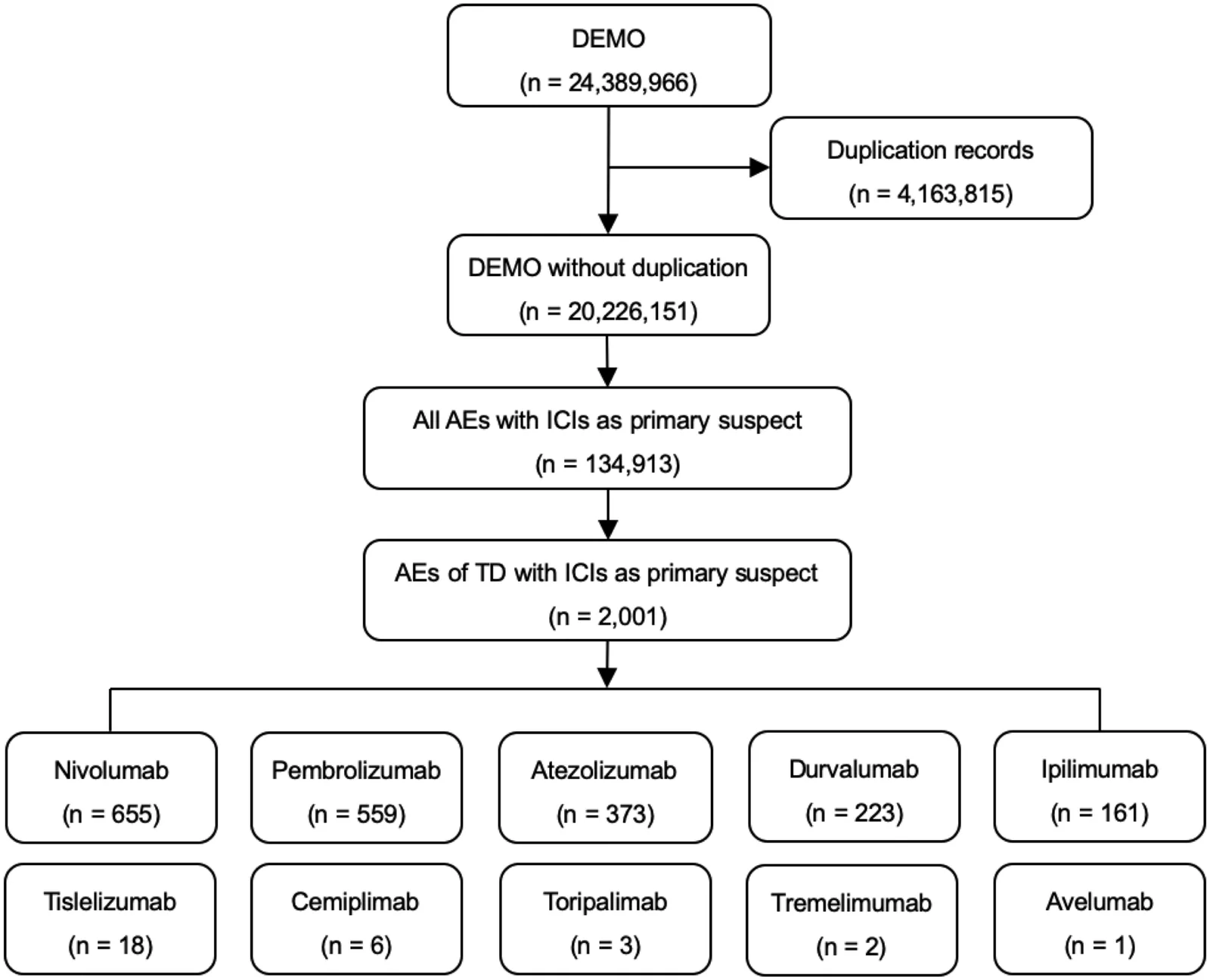
Flow chart depicting the data cleaning process. Data are shown as the number of reports. DEMO, demographic and administrative information; AEs, adverse events; ICIs, immune checkpoint inhibitors; TD, thyroid dysfunction.

The FAERS database contained 24,389,966 reports from January 1^st^ 2014 to March 31^st^ 2026, of which 20,226,151 reports were retained after data deduplication. We identified 134,913 reports with ICIs as primary suspect, of which 2,001 reports were AEs of ICI-TD (**Figure 1**). As the detailed demographic features of patients listed in **Table 3**, a total of 1,758 patients and 10 ICIs were related with ICI-TD during treatment of lung cancer. The leading three ICIs in the number of patients were nivolumab (n = 583), pembrolizumab (n = 504), and atezolizumab (n = 320). In general, ICI-TD was mainly reported in male patients (n = 959, 54.55%) and patients aged ≥ 65 years (n = 856, 48.69%; median [Q1, Q3] = 67 [60, 73]). Most reports were attributed by Asian countries (n = 909, 51.71%), and physicians emerged as the predominant reporter type (n = 892, 50.74%). Reports of ICI-TD from 2014 to 2026 demonstrated a generally ascending tendency, and the vast majority of patients were reported as having serious reaction outcome (n = 1,690, 96.13%).

**Table 3:** Demographic characteristics of patients experiencing ICI-TD.

Characteristics		PD-1					PD-L1			CTLA-4		Total
		Nivolumab	Pembrolizumab	Tislelizumab	Cemiplimab	Toripalimab	Atezolizumab	Durvalumab	Avelumab	Ipilimumab	Tremelimumab	
Number of patients		583	504	16	6	3	320	193	1	130	2	1,758
Gender												
	Female	198 (33.96)	175 (34.72)	0 (0.00)	5 (83.33)	0 (0.00)	70 (21.88)	62(32.12)	1 (100.0)	48 (36.92)	1(50.00)	560 (31.85)
	Male	347 (59.52)	306 (60.71)	0 (0.00)	1 (16.67)	0 (0.00)	106 (33.13)	120(62.18)	0 (0.00)	78 (60.00)	1(50.00)	959 (54.55)
	Unknown^a^	38 (6.52)	23 (4.56)	16 (100.0)	0 (0.00)	3 (100.0)	144 (45.00)	11 (5.70)	0 (0.00)	4 (3.08)	0 (0.00)	239 (13.59)
Age												
	< 18	0 (0.00)	0 (0.00)	0 (0.00)	0 (0.00)	0 (0.00)	0 (0.00)	0 (0.00)	0 (0.00)	0 (0.00)	0 (0.00)	0 (0.00)
	18-44	14 (2.40)	8 (1.59)	0 (0.00)	0 (0.00)	0 (0.00)	1 (0.31)	3 (1.55)	0 (0.00)	2 (1.54)	0 (0.00)	28 (1.59)
	45-64	197 (33.79)	140 (27.78)	3(18.75)	0 (0.00)	0 (0.00)	67 (20.94)	67 (34.72)	1 (100.0)	45 (34.62)	1(50.00)	521(29.64)
	≥ 65	271 (46.48)	296 (58.73)	6(37.50)	4 (66.67)	0 (0.00)	105 (32.81)	94 (48.70)	0 (0.00)	79 (60.77)	1(50.00)	856 (48.69)
	Unknown^a^	101 (17.32)	60 (11.90)	7(43.75)	2 (33.33)	3 (100.0)	147 (45.94)	29 (15.03)	0 (0.00)	4 (3.08)	0 (0.00)	353 (20.08)
	Median (Q1, Q3)	66 (60,72)	69 (62,74)	70 (60,78)	70 (68,76.5)	- ^b^	68 (60,74)	66.5 (60,73)	56 (56,56)	69 (60,75)	63 (59,67)	67 (60,73)
Geographical distribution												
	Asia	206 (35.33)	267(52.98)	16 (100.0)	0 (0.00)	3 (100.0)	197 (61.56)	107(55.44)	0 (0.00)	111 (85.38)	2 (100.0)	909 (51.71)
	Europe	234 (40.14)	140(27.78)	0 (0.00)	4 (66.67)	0 (0.00)	83 (25.94)	55(28.50)	1 (100.0)	11 (8.46)	0 (0.00)	528 (30.03)
	North America	134 (22.98)	92(18.25)	0 (0.00)	2 (33.33)	0 (0.00)	37 (11.56)	26(13.47)	0 (0.00)	8 (6.15)	0 (0.00)	299 (17.01)
	South America	5 (0.86)	4 (0.79)	0 (0.00)	0 (0.00)	0 (0.00)	2 (0.63)	2 (1.04)	0 (0.00)	0 (0.00)	0 (0.00)	13 (0.74)
	Oceania	2 (0.34)	1 (0.20)	0 (0.00)	0 (0.00)	0 (0.00)	1 (0.31)	3 (1.55)	0 (0.00)	0 (0.00)	0 (0.00)	7 (0.40)
	Unknown^a^	2 (0.34)	0 (0.00)	0 (0.00)	0 (0.00)	0 (0.00)	0 (0.00)	0 (0.00)	0 (0.00)	0 (0.00)	0 (0.00)	2 (0.11)
Reporter type												
	Consumer	112 (19.21)	168 (33.33)	0 (0.00)	2 (33.33)	0 (0.00)	7 (2.19)	13 (6.74)	0 (0.00)	4 (3.08)	0 (0.00)	306 (17.41)
	Physician	262 (44.94)	217 (43.06)	3 (18.75)	3 (50.00)	1 (33.33)	174 (54.38)	117 (60.62)	1 (100.0)	112 (86.15)	2 (100.0)	892 (50.74)
	Pharmacist	102 (17.50)	96 (19.05)	4 (25.00)	1 (16.67)	2 (66.67)	125 (39.06)	40 (20.73)	0 (0.00)	12 (9.23)	0 (0.00)	382 (21.73)
	Other health-professional	106 (18.18)	23 (4.56)	0 (0.00)	0 (0.00)	0 (0.00)	5 (1.56)	2 (1.04)	0 (0.00)	2 (1.54)	0 (0.00)	138 (7.85)
	Unknown^a^	1 (0.17)	0 (0.00)	9 (56.25)	0 (0.00)	0 (0.00)	9 (2.81)	21(10.88)	0 (0.00)	0 (0.00)	0 (0.00)	40 (2.28)
Reporting year												
	2014	0 (0.00)	1 (0.20)	0 (0.00)	0 (0.00)	0 (0.00)	0 (0.00)	0 (0.00)	0 (0.00)	0 (0.00)	0 (0.00)	1 (0.06)
	2015	21 (3.60)	1 (0.20)	0 (0.00)	0 (0.00)	0 (0.00)	0 (0.00)	0 (0.00)	0 (0.00)	2 (1.54)	0 (0.00)	24 (1.37)
	2016	66 (11.32)	3 (0.60)	0 (0.00)	0 (0.00)	0 (0.00)	3 (0.94)	0 (0.00)	0 (0.00)	0 (0.00)	0 (0.00)	72 (4.10)
	2017	119 (20.41)	20 (3.97)	0 (0.00)	0 (0.00)	0 (0.00)	4 (1.25)	1 (0.52)	0 (0.00)	4 (3.08)	0 (0.00)	148 (8.42)
	2018	105 (18.01)	76 (15.08)	0 (0.00)	0 (0.00)	0 (0.00)	7 (2.19)	9 (4.66)	0 (0.00)	0 (0.00)	0 (0.00)	197 (11.21)
	2019	74 (12.69)	75 (14.88)	0 (0.00)	0 (0.00)	0 (0.00)	13 (4.06)	22 (11.40)	0 (0.00)	1 (0.77)	0 (0.00)	185 (10.52)
	2020	50 (8.58)	62 (12.30)	0 (0.00)	0 (0.00)	0 (0.00)	20 (6.25)	31 (16.06)	0 (0.00)	2 (1.54)	0 (0.00)	165 (9.39)
	2021	48 (8.23)	47 (9.33)	0 (0.00)	0 (0.00)	0 (0.00)	56 (17.50)	20 (10.36)	1 (50.00)	1 (0.77)	0 (0.00)	173 (9.84)
	2022	25 (4.29)	45 (8.93)	0 (0.00)	0 (0.00)	0 (0.00)	52 (16.25)	22 (11.40)	0 (0.00)	30 (23.08)	0 (0.00)	174 (9.90)
	2023	24 (4.12)	51 (10.12)	0 (0.00)	1 (16.67)	0 (0.00)	46 (14.38)	20 (10.36)	0 (0.00)	26 (20.00)	1 (50.00)	169 (9.61)
	2024	15 (2.57)	63 (12.50)	7 (43.75)	1 (16.67)	0 (0.00)	50 (15.63)	36 (18.65)	0 (0.00)	23 (17.69)	1 (50.00)	196 (11.15)
	2025	29 (4.97)	46 (9.13)	9 (56.25)	3 (50.00)	2 (66.67)	55 (17.19)	27 (13.99)	0 (0.00)	21 (16.15)	0 (0.00)	192 (10.92)
	2026	7 (1.20)	14 (2.78)	0 (0.00)	1 (16.67)	1 (33.33)	14 (4.38)	5 (2.59)	0 (0.00)	20 (15.38)	0 (0.00)	62 (3.53)
Reaction outcome												
	Serious	554 (95.03)	478 (94.84)	16 (100.00)	6 (100.00)	3 (100.00)	314 (98.13)	186(96.37)	1 (100.00)	130 (100.00)	2 (100.00)	1,690 (96.13)
	Non-serious	29 (4.97)	26 (5.16)	0 (0.00)	0 (0.00)	0 (0.00)	6 (1.88)	7 (3.63)	0 (0.00)	0 (0.00)	0 (0.00)	68 (3.87)

Data are shown as the number of patients and percentage.

^a^ “Unknown” denotes missing data.

^b^ “-” denotes that the calculation cannot be mathematically performed due to missing values.

PD-1, anti-programmed cell death receptor-1; PD-L1, anti-programmed death ligand-1; CTLA-4, anti-cytotoxic T-lymphocyte-associated antigen 4; Q1, lower quartile; Q3, upper quartile.

### 3.2 Disproportionality analysis

**Figure 2:**
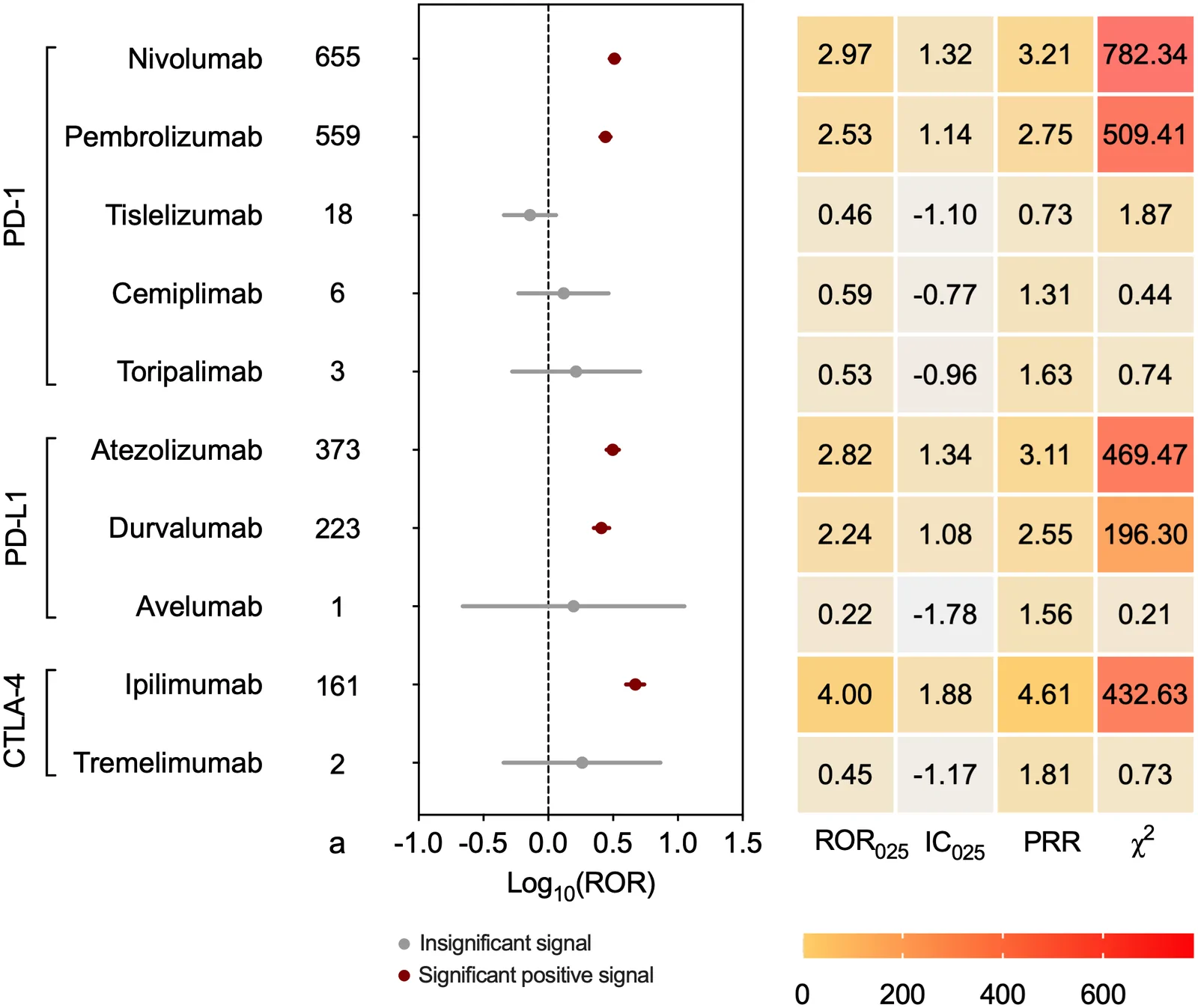
The forest plot showing the pharmacovigilance metrics of the ICI-TD in disproportionality analysis. The ROR were adjusted into logarithms base 10 values. The figure displays adjusted ROR (points) with 95% CI (lines), and the dashed line denotes log_10_(ROR) = 0. A pharmacovigilance signal was considered significant when log_10_(ROR_025_) > 0. a, the number of reports; PD-1, anti-programmed cell death receptor-1; PD-L1, anti-programmed death ligand-1; CTLA-4, anti-cytotoxic T-lymphocyte-associated antigen 4; ROR, reporting odds ratio; ROR _025_, the lower limit of the 95% CI of the ROR; 95% CI, 95% confidence interval; IC, information component; IC_025_, the lower limit of the 95% CI of the IC; PRR, proportional reporting ratio; χ^2^, chi-squared.

This study employed three widely applied disproportionality analysis methods for detecting signals of ICI-TD, namely ROR, PRR, and BCPNN. **Figure 2** exhibits the pharmacovigilance metrics of the ICI-TD identified from all the 2,001 reports. The ROR values were transformed to logarithms base 10 considering readability of the graph. The leading three ICIs in the number of reports were nivolumab (a = 655), pembrolizumab (a = 559), and atezolizumab (a = 373). Furthermore, we found similar patterns of results in pharmacovigilance metrics across the three algorithms. In PD-1 inhibitors, the ICIs that showed significant positive signals in three algorithms were nivolumab (ROR_025_ = 2.97, IC_025_ = 1.32, PRR = 3.21, χ^2^ = 782.34) and pembrolizumab (ROR_025_ = 2.53, IC_025_ = 1.14, PRR = 2.75, χ^2^ = 509.41). In PD-L1 inhibitors, atezolizumab (ROR_025_ = 2.82, IC_025_ = 1.34, PRR = 3.11, χ^2^ = 469.47) and durvalumab (ROR_025_ = 2.24, IC_025_ = 1.08, PRR = 2.55, χ^2^ = 196.30) were the ICIs with positive signal. In the category of CTLA-4, ipilimumab (ROR_025_ = 4.00, IC_025_ = 1.88, PRR = 4.61, χ^2^ = 432.63) displayed a positive AE signal.

In the PT level, “hypothyroidism” was the ICI-TD with the greatest number of reports (a = 901, ROR_025_ = 4.83, IC_025_ = 1.22, PRR = 5.36, χ^2^ = 1,138.77), followed by “hyperthyroidism” (a = 388, ROR_025_ = 3.71, IC_025_ = 1.04, PRR = 4.32, χ^2^ = 402.77) and “thyroid disorder” (a = 198, ROR_025_ = 7.10, IC_025_ = 1.30, PRR = 9.43, χ^2^ = 356.00). There were 9 PTs that were identified significant in all three algorithms, including “hypothyroidism”, “hyperthyroidism”, “thyroid disorder”, “thyroiditis”, “immune-mediated hypothyroidism”, “autoimmune thyroiditis”, “immune-mediated thyroiditis”, “immune-mediated hyperthyroidism”, and “autoimmune hypothyroidism”. The detailed pharmacovigilance metrics of the PTs were listed in **Table 4**.

**Table 4:** Pharmacovigilance metrics of the PT in disproportionality analysis.

PT	a	ROR	ROR_025_	ROR_075_	PRR	χ^2^	IC	IC_025_	IC_075_
Hypothyroidism	901	5.39	4.83	6.02	5.36	1,138.77	1.35	1.22	1.47
Hyperthyroidism	388	4.33	3.71	5.07	4.32	402.77	1.23	1.04	1.41
Thyroid disorder	198	9.44	7.10	12.56	9.43	356.00	1.59	1.30	1.84
Thyroiditis	184	12.36	8.90	17.18	12.35	370.50	1.67	1.37	1.94
Immune-mediated hypothyroidism	139	6.52	4.84	8.78	6.52	202.47	1.44	1.10	1.74
Autoimmune thyroiditis	65	11.30	6.62	19.27	11.29	126.41	1.65	1.11	2.06
Immune-mediated thyroiditis	51	9.42	5.37	16.51	9.41	91.58	1.59	0.98	2.05
Immune-mediated hyperthyroidism	29	3.17	1.88	5.36	3.17	20.79	1.03	0.31	1.61
Thyrotoxic crisis	14	–	–	–	–	41.34	1.98	0.59	2.70
Autoimmune hypothyroidism	14	3.76	1.71	8.28	3.76	12.47	1.15	0.05	1.92
Thyroid hormones decreased	8	2.62	1.01	6.80	2.62	4.26	0.90	-0.48	1.87
Autoimmune thyroid disorder	7	10.34	2.15	49.75	10.34	13.12	1.62	-0.19	2.55
Atrophic thyroiditis	2	–	–	–	–	5.91	1.98	-1.59	3.13
Hypothyroid myopathy	1	–	–	–	–	2.95	1.98	-2.48	3.29
Thyroid atrophy	0	0.00	–	–	0.00	0.68	–	-4.15	2.52
Thyroid dermatopathy	0	0.00	–	–	0.00	0.34	–	-4.12	2.94

“-” denotes that the calculation cannot be mathematically performed according to the formulas in **Table 2**.

PT, preferred terminology; a, the number of reports; ROR, reporting odds ratio; ROR _025_, the lower limit of the 95% CI of the ROR; ROR _075_, the upper limit of the 95% CI of the ROR; 95% CI, 95% confidence interval; PRR, proportional reporting ratio; χ^2^, chi-squared; IC, information component; IC_025_, the lower limit of the 95% CI of the IC; IC_075_, the upper limit of the 95% CI of the IC.

The pharmacovigilance metrics of the positive PTs for each ICI are demonstrated in **Table 5**, with a total of 31 PTs among 5 ICIs. In general, “hypothyroidism” was the most frequently reported ICI-TD among all ICIs. The pembrolizumab had the largest variety of ICI-TD detected (n = 9), followed by ipilimumab (n = 8) and nivolumab (n = 6). The PTs of “autoimmune hypothyroidism” (a = 7, ROR_025_ = 1.96, IC_025_ = 0.33, PRR = 4.70, χ^2^ = 14.67) and “thyroid hormones decreased” (a = 6, ROR_025_ = 2.44, IC_025_ = 0.44, PRR = 6.59, χ^2^ = 18.41) were only identified in the use of pembrolizumab.

**Table 5:** Pharmacovigilance metrics of the positive PT in each ICI.

ICI	PT	a	ROR	ROR_025_	ROR_075_	PRR	χ^2^	IC	IC_025_	IC_075_
Atezolizumab	Hypothyroidism	195	3.59	3.09	4.18	3.57	311.33	1.68	1.44	1.85
	Hyperthyroidism	80	3.08	2.44	3.89	3.07	98.28	1.48	1.11	1.75
	Thyroid disorder	46	4.74	3.44	6.52	4.73	111.46	1.98	1.49	2.33
	Thyroiditis	33	3.73	2.58	5.39	3.72	56.28	1.69	1.11	2.10
Durvalumab	Hypothyroidism	119	2.99	2.48	3.61	2.98	143.41	1.48	1.18	1.70
	Hyperthyroidism	55	2.95	2.24	3.89	2.94	64.63	1.45	1.00	1.77
	Thyroid disorder	16	2.10	1.27	3.48	2.10	8.62	0.98	0.13	1.56
	Thyroiditis	15	2.25	1.33	3.80	2.25	9.76	1.06	0.19	1.67
Ipilimumab	Hypothyroidism	50	3.05	2.30	4.05	3.03	65.81	1.54	1.07	1.87
	Thyroiditis	26	10.55	7.01	15.88	10.51	198.35	3.02	2.37	3.49
	Immune-mediated hypothyroidism	26	12.11	8.02	18.29	12.06	229.97	3.17	2.51	3.63
	Hyperthyroidism	23	2.99	1.97	4.53	2.98	29.26	1.48	0.79	1.98
	Thyroid disorder	14	4.66	2.72	7.98	4.65	37.94	1.99	1.09	2.61
	Immune-mediated hyperthyroidism	9	15.66	7.67	31.96	15.64	103.52	3.01	1.87	3.77
	Immune-mediated thyroiditis	8	11.09	5.30	23.21	11.07	64.57	2.70	1.48	3.50
	Thyrotoxic crisis	3	22.28	6.22	79.89	22.27	47.89	2.39	0.32	3.59
Nivolumab	Hypothyroidism	309	3.30	2.91	3.74	3.28	382.67	1.47	1.28	1.61
	Hyperthyroidism	140	3.16	2.62	3.81	3.15	161.89	1.42	1.14	1.62
	Thyroid disorder	66	3.94	2.98	5.20	3.93	107.65	1.65	1.24	1.94
	Thyroiditis	66	4.71	3.54	6.28	4.71	136.96	1.83	1.42	2.13
	Autoimmune thyroiditis	26	5.37	3.37	8.55	5.36	63.05	1.91	1.26	2.38
	Thyrotoxic crisis	5	6.42	2.15	19.15	6.42	14.70	1.77	0.21	2.75
Pembrolizumab	Hypothyroidism	211	2.16	1.86	2.50	2.15	110.36	0.98	0.75	1.14
	Immune-mediated hypothyroidism	81	8.10	6.11	10.74	8.09	301.45	2.35	1.99	2.62
	Thyroid disorder	52	3.02	2.23	4.10	3.02	56.24	1.37	0.90	1.70
	Thyroiditis	42	2.73	1.95	3.82	2.73	37.51	1.25	0.73	1.61
	Immune-mediated thyroiditis	28	8.68	5.34	14.10	8.67	110.65	2.34	1.71	2.79
	Autoimmune thyroiditis	22	4.43	2.72	7.22	4.43	42.75	1.73	1.02	2.23
	Immune-mediated hyperthyroidism	15	4.42	2.45	7.99	4.42	29.06	1.70	0.83	2.30
	Autoimmune hypothyroidism	7	4.70	1.96	11.25	4.70	14.67	1.64	0.33	2.49
	Thyroid hormones decreased	6	6.59	2.44	17.82	6.59	18.41	1.85	0.44	2.76

PT, preferred terminology; ICI, immune checkpoint inhibitor; a, the number of reports; ROR, reporting odds ratio; ROR _025_, the lower limit of the 95% CI of the ROR; ROR _075_, the upper limit of the 95% CI of the ROR; 95% CI, 95% confidence interval; PRR, proportional reporting ratio; χ^2^, chi-squared; IC, information component; IC_025_, the lower limit of the 95% CI of the IC; IC_075_, the upper limit of the 95% CI of the IC.

### 3.3 TTO analysis

**Figure 3:**
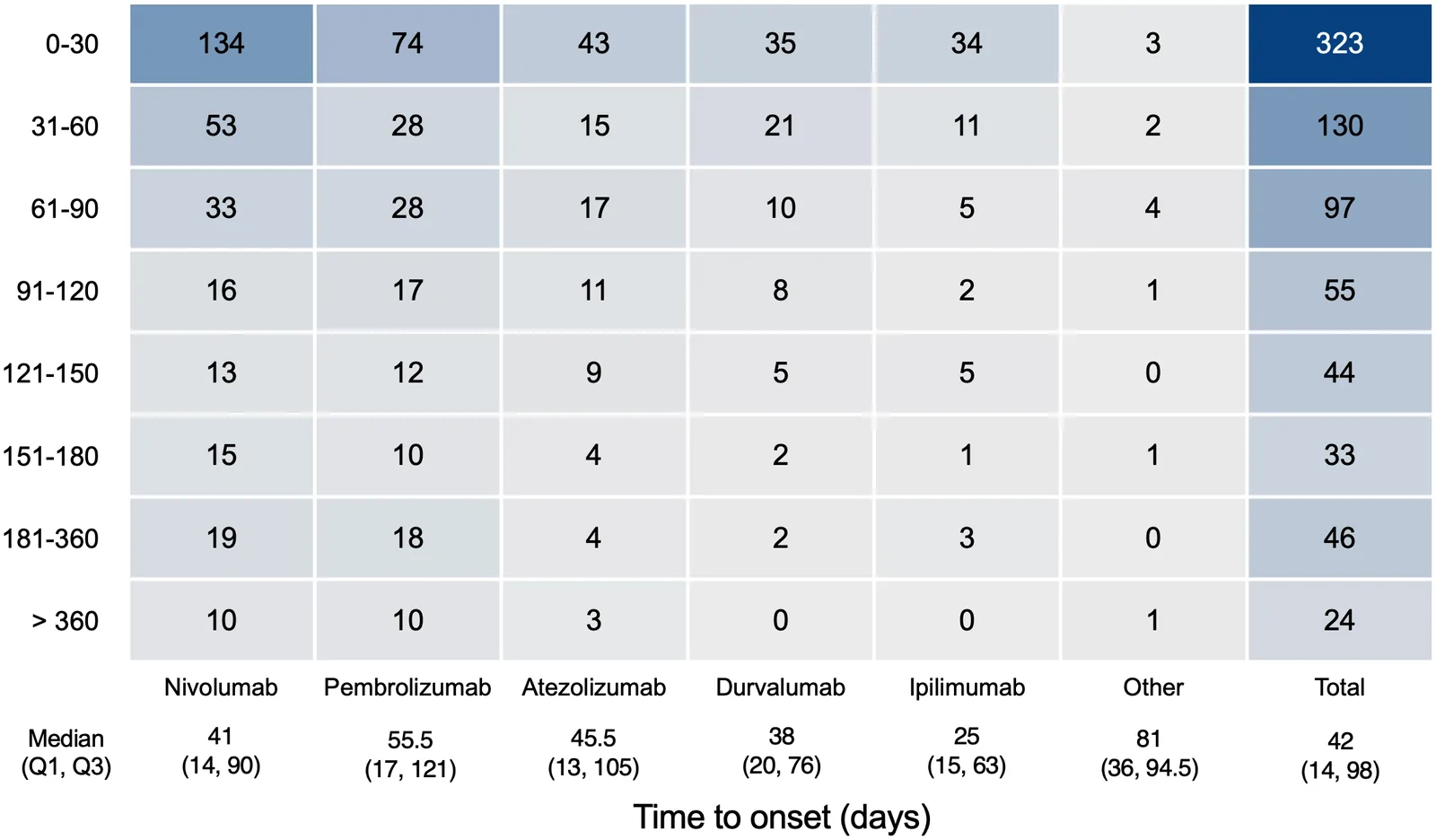
The distribution patterns of TTO among all ICIs. Data are shown as the number of patients with valid TTO values. The ICIs with valid TTO number < 5, including tislelizumab (n = 4), toripalimab (n = 3), cemiplimab (n = 2), tremelimumab (n = 2), and avelumab (n = 1), were integrated under the column of “Other” considering readability of the data. TTO, time-to-onset; Q1, lower quartile; Q3, upper quartile.

We identified a total of 752 patients with valid TTO values and included them into the final analysis. There were certain ICIs had valid TTO number < 5, including tislelizumab (n = 4), toripalimab (n = 3), cemiplimab (n = 2), tremelimumab (n = 2), and avelumab (n = 1). Due to the limited sample size of the above-mentioned ICIs and the insufficient data reliability caused (27), these 5 ICIs were grouped under the column of “Other” in **Figure 3**. Overall, we found that the median TTO of ICI-TD was 42 days [Q1, Q3] = [14, 98], and ICI-TD was predominantly observed within 30 days after ICI administration (n = 323, 42.95% of all 752 patients). Among all ICIs, ipilimumab had the smallest TTO median value (median, [Q1, Q3] = 25 [15, 63]), whereas pembrolizumab had the largest TTO median value (median, [Q1, Q3] = 55.5 [17, 121]).

**Figure 4:**
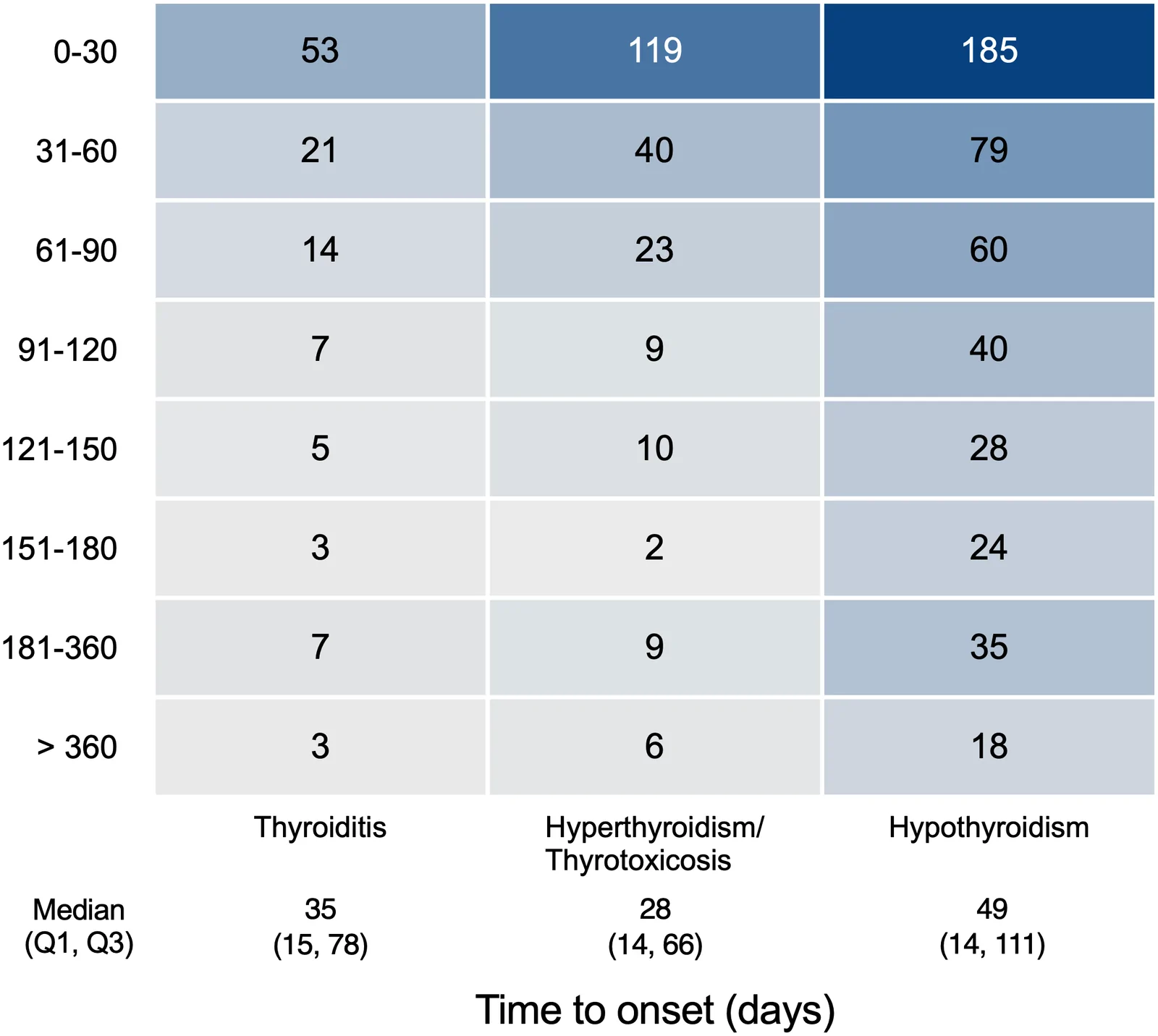
The distribution patterns of TTO in PT subgroups. Data are shown as the number of valid TTO values, and some patients might contain more than one PT. Detailed list of the three PT subgroups is as follows: thyroiditis (including “atrophic thyroiditis”, “autoimmune thyroiditis”, “immune-mediated thyroiditis”, and “thyroiditis”), hyperthyroidism/thyrotoxicosis (including “hyperthyroidism”, “immune-mediated hyperthyroidism”, and “thyrotoxic crisis”), and hypothyroidism (including “autoimmune hypothyroidism”, “hypothyroid myopathy”, “hypothyroidism”, “immune-mediated hypothyroidism”, and “thyroid hormones decreased”). TTO, time-to-onset; Q1, lower quartile; Q3, upper quartile.

Subsequently, we performed an exploratory TTO analysis of PT subgroups, which mainly included the following three categories: thyroiditis (including “atrophic thyroiditis”, “autoimmune thyroiditis”, “immune-mediated thyroiditis”, and “thyroiditis”), hyperthyroidism/thyrotoxicosis (including “hyperthyroidism”, “immune-mediated hyperthyroidism”, and “thyrotoxic crisis”), and hypothyroidism (including “autoimmune hypothyroidism”, “hypothyroid myopathy”, “hypothyroidism”, “immune-mediated hypothyroidism”, and “thyroid hormones decreased”). The PTs of “autoimmune thyroid disorder”, “thyroid atrophy”, “thyroid dermatopathy”, and “thyroid disorder” were excluded from the categories due to the ambiguous/overlapping nature of these PTs. A total of 800 valid TTO values were included in the analysis (some patients might contain more than one PT). We found that PTs were predominantly observed within 30 days after ICI administration (n = 357, 44.63% of all 800 valid TTO values). The category of “hyperthyroidism/thyrotoxicosis” had the smallest TTO median value (median, [Q1, Q3] = 28 [14, 66]), whereas the category of “hypothyroidism” had the largest TTO median value (median, [Q1, Q3] = 49 [14, 111]) (**Figure 4**).

## 4 Discussion

To our knowledge, this is the first study employed real-world evidence from the FAERS database to comprehensively mine the clinical characteristics of ICI-TD during lung cancer treatment. Building on prior research (10, 12, 13), the present study addressed the current limitations through a longer research timespan (January 1^st^ 2014 to March 31^st^ 2026) and expanded list of drugs (10 ICIs). Our findings also complemented clinical trial data of ICI-TD from the perspective of pharmacovigilance, enhancing the clinical understanding of ICI-TD during lung cancer treatment.

In demographic analysis, our results indicated a higher proportion of male patients, contrasting with prior evidence suggesting higher ICI-TD reporting rate in female patients (10, 28). Such difference might be partially attributed to the higher proportion of males in lung cancer population (1), yet the gender susceptibility difference warrants further validation in real-world cohort studies. We also found a higher reporting rate of elderly patients experiencing ICI-TD, which was consistent with previous pharmacovigilance findings (10, 12, 13). Meanwhile, we should acknowledge that although most patients were reported to have serious outcome, this might reflect the clinical condition and hospitalization status of cancer patients rather than the intrinsic severity of ICI-TD.

In disproportionality analysis, our study showed higher reporting frequency of “hypothyroidism” in all ICIs, which echoed previous pharmacovigilance findings (10, 13) and clinical-derived data (29, 30). However, since the initial phase of hyperthyroidism is often transient and asymptomatic, while hypothyroidism is more persistent and symptomatic (31), the potential reporting bias cannot be ignored. On the other hand, hypothyroidism and hyperthyroidism are considered as consecutive stages of thyroiditis rather than distinct diseases, manifesting as hyperthyroidism followed by hypothyroidism (32, 33). This might partly responsible for the relatively low number of “thyroiditis” and “thyroid disease” in our results.

TTO analysis is considered as a critical approach in the prediction and identification of AEs (34). As a consequence of the large number of absent or invalid TTO entries in the database, only 42.78% of all data (752 of 1,758 patients) met the inclusion criteria. We found that the median TTO of ICI-TD was 42 days following ICIs administration, whereas a considerable number of patients experienced ICI-TD after 6 months. It is recommended that clinicians should perform more frequent thyroid function surveillance in the first 3 months of treatment, and long-term follow-up assessment is required (35). Previous findings from a TTO analysis with PT subgroups found that the TTO of “hyperthyroidism” was earlier than that of “hypothyroidism” in patients treated with nivolumab, pembrolizumab, and ipilimumab (13), which is consistent with the aforementioned temporal pattern of thyroiditis. Our results of PT subgroup analysis were consistent with previous findings that the TTO value of “hyperthyroidism/thyrotoxicosis” was smaller than that of “hypothyroidism”. However, we still need to emphasize that due to the limited sample size in the PT subgroup analysis, the robustness of the results might be affected. Additionally, we performed an exploratory comparative analysis of demographic characteristics between patients with and without TTO values. Interestingly, we found significant differences in gender (*P* = 0.001), geographical distribution (*P* < 0.001), reporter type (*P* < 0.001), reporting year (*P* < 0.001), and reaction outcome (*P* < 0.001) (**Supplementary Table 2**). These findings suggested that TTO data were not randomly lost, but rather indicative of potential selection bias. The direction of this bias cannot be determined from the available data because the TTO values were missing in one group and could not be compared. Such bias might potentially limit the representativeness of TTO results for the overall dataset. Moreover, since the absence of original data was mainly related to reporting behavior, it cannot be adequately corrected by subsequent data supplementation or statistical methods. We therefore emphasize that our TTO findings should be interpreted with caution. Follow-up studies should establish standardized AE reporting guidelines to minimize the absence of key information and improve the reliability of real-world pharmacovigilance data.

There were several limitations existed in this study. First, the reliance of FAERS database on voluntary reporting inevitably leads to challenges of missing data, under reporting, and delayed reporting, which led to insufficient comprehensiveness of the data. Therefore, we should acknowledge that these results remain exploratory and require further validation with larger datasets. Second, due to the absence of detailed medical records, such as medical history, comorbidities, and concurrent medications (36), the findings only enabled us to determine drug-AE reporting association rather than incidence, clinical risk, or causality. Finally, we only explored the primary suspected ICIs and did not discriminate between ICI monotherapy and combination therapy. Thus, the potential confounding effects of combination therapy should be considered when interpreting the findings. Future studies in multiple strategies, such as active surveillance systems to reduce underreporting and reporting delays (37, 38), prescription and medical records data to estimate exposure denominators (39), and machine learning algorithms to detect complex pharmacovigilance signals (40, 41), would provide a more comprehensive assessment of AEs in future studies.

## 5 Conclusions

This pharmacovigilance study systematically analyzed the demographic characteristics and pharmacovigilance signal patterns of ICI-TD in patients with lung cancer via real-world data from the FAERS database. Our findings would offer a referential perspective for the clinical understanding of the pharmacovigilance signals of ICI-TD.

## Data Availability

The original contributions presented in the study are included in the article/**Supplementary Material**. Further inquiries can be directed to the corresponding author.
