# Supplementary material for: Immune checkpoint inhibitor-related thyroid dysfunction during treatment of lung cancer: a disproportionality analysis based on the FDA adverse event reporting system
Source: Front Immunol. 2026 Sep 9;17:1902939. doi: 10.3389/fimmu.2026.1902939 (PMC13597272; doi:10.3389/fimmu.2026.1902939)
Supplement: Supplementary file 1 [file SupplementaryFile1.docx]

**Supplementary Materials**

**Table S1.** The READUS-PV checklist.

| **Section and topic** | **Item #** | **Checklist item** | **Location where item is reported** |
| --- | --- | --- | --- |
| **Title** |  |  |  |
|  | *1a* | *If disproportionality analyses are a prominent component of the published study, the study should be identified as a “disproportionality analysis”. The type of data and name of the database(s) should be specified.* | *Page 1* |
|  | *1b* | *Report the name of adverse event(s) and/or drug(s) under study, when applicable.* | *Page 1* |
| **Introduction** |  |  |  |
| Background | *2a* | *Describe the drug(s) and its utilization, the nature of the adverse event(s) under study and its frequency, and the existing knowledge on the drug-event combination.* | *Page 4-5* |
|  | *2b* | *Specify the rationale for performing the analysis, e.g., as part of routine pharmacovigilance, to investigate an overall safety profile, or to assess a pre-specified hypothesis.* | *Page 4-5* |
|  | *2c* | *Explain why ICSR databases and disproportionality analysis are suitable to fill the knowledge gap.* | *Page 4-5* |
| Objectives | *3* | *State specific objectives, identifying the adverse event(s), the drug(s), and the reference group, including any pre-specified hypothesis, if applicable.* | *Page 5* |
| **Methods** |  |  |  |
| Study design | *4a* | *Identify the study (i.e., “disproportionality analysis”) and the type of data used (e.g., “individual case safety reports”).* | *Page 6-8* |
|  | *4b* | *Provide an outline of the entire study design, including primary and sensitivity analyses performed, and other designs such as case-by-case analysis or literature review.* | *Page 6-8*  *Figure 1* |
| Data description, access, and pre-processing | *5a* | *Specify the name of the database(s), the database(s) custodian, and the coverage. Specify the type/number of drugs included within the database and the thesaurus, taxonomies, or ontologies used for coding drugs and events.* | *Page 6-8* |
|  | *5b* | *Specify the extraction dates and describe and justify all choices used for data pre-processing, including any data transformation or exclusion, if appropriate.* | *Page 6-8* |
| Variables definition | *6a* | *Describe the study population, including any restriction.* | *Page 6-8* |
|  | *6b* | *Describe the nature and the meaning of key variables assessed in the work.* | *Page 6-8* |
|  | *6c* | *Specify and justify any grouping of drugs or events. For drugs, specify and justify whether active ingredients/trade names/salts were considered and/or the selected role.* | *Page 6-8* |
|  | *6d* | *Describe any additional data source used, the type of data, and how they interact with ICSRs.* | *Not applicable* |
| Statistical methods | *7a* | *Present any descriptive analysis performed, specifying variables investigated, statistical tests, and significance thresholds.* | *Page 8-9* |
|  | *7b* | *Describe the measure(s) selected for the disproportionality analysis including any threshold used to identify signals of disproportionate reporting. Explain the reason for this choice if applicable.* | *Table 1, Table 2* |
|  | *7c* | *Clearly describe any sensitivity analysis and any tool to control confounding, including any restriction, subgroup, stratification, adjustment, or interaction.* | *Page 8-9* |
|  | *7d* | *Specify the variables and methods used for the case-by-case analysis, including any algorithm or criteria used to assess causality, if performed.* | *Not applicable* |
|  | *7e* | *Specify any statistical methods used for other data sources.* | *Not applicable* |
| **Results** |  |  |  |
| Participants | *8a* | *Specify the number of individual case safety reports included at each stage, including reasons for exclusion.* | *Page 9-11* |
|  | *8b* | *Provide key demographic and clinical characteristics of cases, if possible comparing cases with any appropriate reference group.* | *Table 3*  *Page 9-11* |
| Disproportionality analysis | *9* | *Present all results including confidence intervals. Present also results of sensitivity analyses, if performed.* | *Figure 2-3*  *Table 4-5* |
| Case-by-case analysis | *10* | *Present the case-by-case analysis of key variables. Present the causality assessment, if applicable.* | *Not applicable* |
| **Discussion** |  |  |  |
| Key results | *11* | *Discuss key results with reference to study objectives and contextualize them within the current literature and other consulted sources. Clearly discriminate between expected reactions and emerging safety signals.* | *Page 12-14* |
| External validity | *12a* | *Discuss the external validity of the results to the general population.* | *Page 12-14* |
|  | *12b* | *Discuss the potential relevance of results in clinical practice* | *Page 12-14* |
|  | *12c* | *Propose further study designs if applicable* | *Page 12-14* |
| Limitations | *13* | *Present general limitations, making clear that disproportionality analysis alone cannot prove causation or measure incidence, and specific limitations, including confounding and reporting bias and efforts to mitigate them.* | *Page 14* |
| **Declarations** |  |  |  |
|  | *14a* | *Provide the source of funding/sponsorship and the role of the funders/sponsors for the present study and for any original study on which the present article is based.* | *Page 15* |
|  | *14b* | *Clearly identify potential commercial and intellectual conflicts of interest (e.g., link to any drug/event investigated, whether financial, legal action, or software used).* | *Page 15* |
|  | *14c* | *Declare any institutional approval needed or granted in the investigation.* | *Page 15* |
|  | *14d* | *Include a statement on data availability, code availability (including the version of the statistical software used), and protocol registration.* | *Page 15* |

**Table S2.** Comparative analysis of demographic characteristics between patients with and without TTO values.

| Characteristics | | Without TTO values | With TTO values | χ^2^ (*df*) | *P* value |  |
| --- | --- | --- | --- | --- | --- | --- |
| Gender |  |  |  | 10.42 (1) | 0.001 |  |
|  | Female | 319 (31.71) | 241 (32.74) |  |  |  |
|  | Male | 464 (46.12) | 495 (67.26) |  |  |  |
| Age |  |  |  | 1.78 (2) | 0.41 |  |
|  | < 18 | 0 (0.00) | 0 (0.00) |  |  |  |
|  | 18-44 | 16 (2.37) | 12 (1.65) |  |  |  |
|  | 45-64 | 258 (38.17) | 263 (36.08) |  |  |  |
|  | ≥ 65 | 402 (59.47) | 454 (62.28) |  |  |  |
| Geographical distribution | |  |  | 78.96 (4) | < 0.001 |  |
|  | Asia | 461 (45.87) | 448 (59.65) |  |  |  |
|  | Europe | 292 (29.05) | 236 (31.42) |  |  |  |
|  | North America | 237 (23.58) | 62 (8.26) |  |  |  |
|  | South America | 9 (0.90) | 4 (0.53) |  |  |  |
|  | Oceania | 6 (0.60) | 1 (0.13) |  |  |  |
| Reporter type | |  |  | 35.81 (3) | < 0.001 |  |
|  | Consumer | 162 (16.55) | 144 (19.49) |  |  |  |
|  | Physician | 477 (48.72) | 415 (56.16) |  |  |  |
|  | Pharmacist | 267 (27.27) | 115 (15.56) |  |  |  |
|  | Other health-professional | 73 (7.46) | 65 (8.80) |  |  |  |
| Reporting year | |  |  | 65.06 (12) | < 0.001 |  |
|  | 2014 | 0 (0.00) | 1 (0.13) |  |  |  |
|  | 2015 | 9 (0.89) | 15 (1.99) |  |  |  |
|  | 2016 | 37 (3.68) | 35 (4.65) |  |  |  |
|  | 2017 | 72 (7.16) | 76 (10.11) |  |  |  |
|  | 2018 | 87 (8.65) | 110 (14.63) |  |  |  |
|  | 2019 | 96 (9.54) | 89 (11.84) |  |  |  |
|  | 2020 | 85 (8.45) | 80 (10.64) |  |  |  |
|  | 2021 | 106 (10.54) | 67 (8.91) |  |  |  |
|  | 2022 | 93 (9.24) | 81 (10.77) |  |  |  |
|  | 2023 | 104 (10.34) | 65 (8.64) |  |  |  |
|  | 2024 | 137 (13.62) | 59 (7.85) |  |  |  |
|  | 2025 | 139 (13.82) | 53 (7.05) |  |  |  |
|  | 2026 | 41 (4.08) | 21 (2.79) |  |  |  |
| Reaction outcome | |  |  | 25.22 (1) | < 0.001 |  |
|  | Serious | 947 (94.14) | 743 (98.80) |  |  |  |
|  | Non-serious | 59 (5.86) | 9 (1.20) |  |  |  |

Note: Data are shown as the number of patients and percentage. The differences between two groups (i.e., with and without TTO values) were examined using crosstabs analysis and Pearson chi-square analysis. The predetermined α level was 0.05, and a 2-sided *P* value < 0.05 was considered statistically significant.

Abbreviations: TTO, time-to-onset; χ^2^, chi-square; *df*, degrees of freedom.
